# Emerging therapies for hemophilia: controversies and unanswered questions

**DOI:** 10.12688/f1000research.12491.1

**Published:** 2018-04-24

**Authors:** Valder R. Arruda, Bhavya S. Doshi, Benjamin J. Samelson-Jones

**Affiliations:** 1 The Children's Hospital of Philadelphia, Philadelphia, PA, USA; 2 Perelman School of Medicine, University of Pennsylvania, Philadelphia, PA, USA; 3 Raymond G. Perelman Center for Cellular and Molecular Therapeutics, Philadelphia, PA, USA

**Keywords:** hemophilia, EHL, NFT, gene therapy

## Abstract

Several new therapies for hemophilia have emerged in recent years. These strategies range from extended half-life factor replacement products and non-factor options with improved pharmacokinetic profiles to gene therapy aiming for phenotypic cure. While these products have the potential to change hemophilia care dramatically, several challenges and questions remain regarding broader applicability, long-term safety, and which option to pursue for each patient. Here, we review these emerging therapies with a focus on controversies and unanswered questions in each category.

## Introduction

Hemophilia is an X-linked bleeding disorder resulting from deficiency of factor VIII (FVIII) or factor IX (FIX) due to mutations in the *F8* or *F9* genes, respectively. The disorder affects approximately 1 in 10,000 male births worldwide; 80% of cases are of FVIII deficiency or hemophilia A (HA), and 20% are FIX deficiency or hemophilia B (HB).

The disease phenotype is characterized by bleeding into the joints (hemarthrosis), skeletal muscle, soft tissues, and enclosed spaces such as the intracranium and retroperitoneum, which can be fatal. Residual factor level correlates directly with bleeding phenotype wherein patients with severe disease (<1%) present with spontaneous bleeds, those with moderate disease (1–5%) bleed with minor trauma and rarely spontaneously, and those with mild disease (6–30%) bleed only secondary to trauma or invasive procedures. Current treatment includes replacement therapy with plasma-derived (pd) or recombinant (r) clotting factor concentrates either “on demand” for acute bleeding or prophylactically to prevent bleeding. However, in the US, only approximately 60% of young adults and adults report adherence to prophylaxis, and the average cost of the recommended dose of prophylactic therapy is estimated at approximately $200,000–300,000/year ^1^. Owing to the high cost and need for life-long therapy, only 20% of patients worldwide have regular access to treatment.

**Figure 1.**
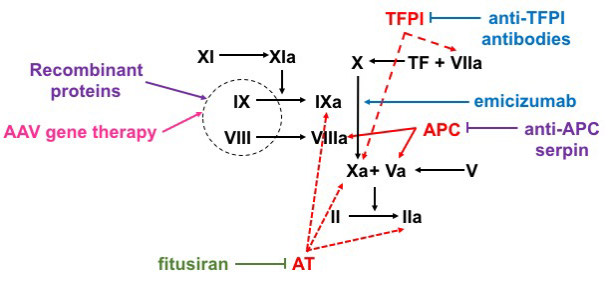
Mechanism of action of hemophilia therapies. Factor X (FX) can be activated to FXa either via FIXa–FVIIIa complex or the tissue factor (TF) factor–FVIIa complex. FXa and FVa activate prothrombin (FII) to thrombin (FIIa) in order to generate a fibrin clot. Natural anti-coagulants targeted by non-factor therapeutics are represented in red. Protein-based therapeutics are represented in purple, nucleotide-based therapeutics are represented in blue, and antibody-based therapeutics are represented in green. Fitusiran decreases the production of antithrombin (AT), decreasing its inhibition of FIXa, FXa, and FIIa. Concizumab and anti-protein C serine protease inhibitors (serpins) block tissue factor pathway inhibitor (TFPI) from inhibiting FXa and TF–FVIIa complex or protein C from inhibiting FVIIIa and FVa, respectively. Emicizumab is a FVIIa mimic that brings together FIXa and FX to generate FXa. Factor-based therapies include adeno-associated virus (AAV)-based liver-directed gene therapy, which results in endogenous factor production, and exogenously given factor therapeutics given intravenously. APC, activated protein C; EHL, extended half-life.

Currently, the most serious complication of hemophilia therapy is the formation of neutralizing alloantibodies (inhibitors) that preclude the hemostatic effect of factor replacement. In HA, 30% of severe patients and 5–10% of non-severe patients develop inhibitors compared to only 3–5% of severe HB patients ^2^. Inhibitors are associated with increased morbidity and mortality, and only a few variably effective and expensive hemostatic options (termed bypassing agents) are available to these patients, such as activated prothrombin complex concentrate (aPCC) and recombinant activated factor VII (rFVIIa). Inhibitors do not respond well to immunosuppressive therapy alone ^3^. The most efficacious and cost-effective treatment for inhibitors is immune tolerance induction (ITI), which consists of frequent injections of FVIII or FIX for extended periods of time. The success rate of inhibitor eradication is 60% and 30% for HA and HB patients, respectively ^4^. Thus, the development of novel strategies that could facilitate prophylaxis for patients with and without inhibitors is needed ( Figure 1).

## Extended half-life products

The half-lives of FVIII and FIX in plasma are 10–12 hours and 16–18 hours, respectively ^5^. For prophylaxis, patients with severe disease need to be injected with standard half-life (SHL) replacement therapy two to three times per week to minimize spontaneous bleeds by maintaining a factor level >1%. Consequently, pharmaceutical development has focused on the optimization of product pharmacokinetics to decrease infusion frequency. Technologies used to create these extended half-life (EHL) products decrease clearance by fusion to the constant fragment (Fc) of IgG or albumin, PEGylation (the covalent attachment of polymeric hydrophilic polyethylene glycol [PEG] molecules), or protein modifications ^6^. Alternative strategies to extend half-life such as carboxy-terminal peptide technology, hydroxyethyl starch, and hyperglycosylation are still in early preclinical phases. Fusion technologies avoid lysosomal degradation of the protein by utilizing the neonatal Fc receptor to salvage factor proteins and recycle them into the circulation ^7^. PEGylation increases half-life by reducing proteolytic cleavage and inhibiting receptor-mediated clearance ^8^.

### Are EHL products “better”?

EHL–rFIX products have successfully decreased infusion frequency from twice weekly to every 10–14 days using fusion to Fc–IgG1 or albumin or PEGylation technologies ^9, 10^; however, EHL–rFVIII products have only decreased infusions from about three to about two times per week ^11– 13^. Efforts are underway to understand why EHL–rFVIII products have not been more successful. Clearance of FVIII from the circulation occurs mostly in complex with von Willebrand factor (VWF); the half-life of VWF ranges from 4–26 hours, with an average of approximately 15 hours ^14^. The prevailing theory is that the half-life of VWF imposes an upper limit on FVIII half-life prolongation. Two recent studies evaluating the biochemical interaction between VWF and FVIII demonstrated that it was dependent upon the VWF D’D3 and FVIII C1 domains ^15, 16^. Preclinical studies are now assessing VWF D’D3 and/or FVIII protein modifications to enhance VWF binding or half-life ^17, 18^. All licensed EHL products recommend tailoring the dose to the individual patient’s pharmacokinetic response. Hence, for each individual patient, a particular product may not offer significant half-life extension and therefore may be less effective from a cost and convenience standpoint. However, EHL products might afford clinicians the ability to customize a patient’s dose and frequency for a higher goal factor trough that minimizes bleeding and maximizes compliance. Indeed, post-hoc analysis of rFVIII–Fc trials demonstrates improved bleeding rate with similar or increased physical activity ^19^ and improved joint arthropathy scores in patients treated with rFVIII–Fc either prophylactically or on demand ^20^. These benefits likely reflect higher troughs and improved compliance secondary to decreased infusion frequency with EHL products.

### Are EHL products safer?

Whether the EHL products alter the immunogenicity profile of rFVIII products and might be safer than pdFVIII–VWF products is an unanswered question. There is only one reported case of inhibitor development to date for EHL products ^21^. Early data support the hypothesis that Fc fusion may potentially decrease immunogenicity in previously treated patients, but analogous data for previously untreated patients are not yet available. To date, seventeen patients with inhibitors have been successfully tolerized with rFVIII–Fc, including some who had previously failed ITI with SHL rFVIII ^22– 24^. The success of this strategy is likely due to a longer time with consistent antigen exposure with rFVIII–Fc ^25^. Furthermore, in murine studies, treatment with rFVIII–Fc expanded tolerance-inducing regulatory T cells ^26^. A clinical trial for immune tolerance therapy with rFVIII–Fc as first-line therapy is ongoing (NCT03093480). The available data suggest that the immune profile of EHL products is similar to, if not better than, SHL recombinant products. There are no data available comparing recombinant EHL with plasma-derived factors.

However, half-life-extending strategies utilizing protein sequence changes in the bypassing agent rFVIIa have proven problematic. Two different EHL rFVIIa products with changes in protein sequence to enhance stability progressed through early trials, but patients in later-phase trials developed inhibitors to the investigational drug that cross-reacted to endogenous FVIIa ^27, 28^. These failures underscore the importance of further characterizing the immune response to novel products, especially with amino acid changes, in order to better predict immunogenicity in patients ^29^. For PEGylated products, long-term follow-up is needed to understand the implication of anti-PEG antibodies and whether significant PEG accumulation occurs, as was demonstrated in animal models ^30^. Currently available data suggest that albumin-fused rFIX provides a similar dosing frequency to PEGylated rFIX without the theoretical concern of PEG accumulation ^9^.

### Does switching product affect safety of treatment?

There are no randomized trial data to understand whether switching a patient from one product to another will change the risk of inhibitor formation. Inhibitors typically occur within the first 50 exposure days to factor in severe patients ^31^. Prior studies done after national health services required a change in FVIII product have found varied results ^32– 36^. Consequently, providers who switch products do so carefully with inhibitor titers pre- and post-switch and generally avoid switching in patients with a history of inhibitor who have been tolerized with a specific product.

The devastating HIV and viral hepatitis epidemics amongst people with hemophilia from viral contamination of plasma-derived products in the 1980’s spurred the development and transition to recombinant products ^37, 38^. However, long-standing debate has ensued in the field regarding the relative immunogenicity of pdFVIII and SHL rFVIII products because of conflicting results of retrospective studies ^39– 42^. Recently, the only prospective, randomized trial (SIPPET) comparing the immunogenicity of VWF-containing pdFVIII products to SHL rFVIII products in HA demonstrated increased immunogenicity with SHL rFVIII (hazard ratio 1.86) ^43^. How best to apply these results in clinical decision making is complicated by the emergence of EHL–rFVIII products since SIPPET. EHL products, especially EHL–rFIX, may increase compliance by decreasing infusion frequencies, but it is unknown if EHL–rFVIII products are more or less immunogenic than SHL rFVIII or pdFVIII products. Although certain factors can guide clinicians to risk of inhibitor formation (e.g. disease severity, underlying mutation, and family history), inhibitor risk stratification is an imprecise science ^44^. Furthermore, in SIPPET subanalysis, use of pdFVIII versus SHL rFVIII surprisingly proved beneficial only for “low”- rather than “high”-risk patients ^45^. SIPPET results also pose a dilemma for clinicians who are hesitant to return to pdFVIII because of the history of prior blood-borne infectious epidemics or the potential for new ones. Though current virucidal techniques to date have successfully mitigated the risk of emerging viruses (e.g. West Nile ^46^ and Zika ^47^ virus), the risk posed by prions ^48^ and other unidentified infectious agents remains unknown. Preliminary data also suggest that EHL products may fare better for ITI **,** both for those who fail with rFVIII and as first-line therapy ^22, 23^.

In conclusion, clinicians face a litany of challenging choices for factor replacement. They are tasked with prioritizing convenience, cost, compliance, and safety without definitive ability to predict immune or product pharmacokinetic responses. Though it is tempting to “move backwards” to pdFVIII based upon SIPPET and improved viral inactivation techniques, the potential of a novel blood-borne pathogen epidemic should engender caution. As inhibitors occur in only 20–30% of severe patients, the economic impact of widespread use of pdFVIII (as implied by recent risk-stratified SIPPET subanalysis ^45^) should be considered as national groups develop guidelines. Further insight into the basic mechanisms underlying FVIII immunogenicity and clearance, the role of VWF in both, and clear risk stratification schema for inhibitor formation will tremendously advance the ability to identify the best product for each patient.

## Non-factor therapies

Novel technologies aimed at promoting hemostasis in patients with hemophilia without replacing the deficient factor are currently in clinical development. These include FVIII mimics ^49– 52^ and agents that obstruct the function of natural anti-coagulants, such as antithrombin (AT) ^53, 54^, tissue factor pathway inhibitor (TFPI) ^55– 59^, and activated protein C (APC) ^60, 61^.

Emicizumab is a bispecific antibody that can simulate the biological function of FVIII sufficiently to produce a pro-coagulant effect in patients with HA ^49– 52^. One antigen-binding fragment (Fab) of the bispecific antibody recognizes activated FIX (FIXa), while the other Fab recognizes its substrate, factor X (FX); the simultaneous binding of FX and FIXa by emicizumab sufficiently orients these factors to facilitate the proteolytic activation of FX by FIXa without FVIIIa cofactor activity. Clinical studies have demonstrated that emicizumab is efficacious in decreasing, though not eliminating, the bleeding rate in HA patients with and without inhibitors ^49, 51^. Encouragingly, the annualized bleeding rate (ABR) of inhibitor patients receiving emicizumab prophylaxis (ABR 2.9) is lower than rates with prophylactic bypassing therapies (ABR 10–36) ^62, 63^. The rationale of targeting natural anti-coagulants is based on clinical observations ^64– 67^ and animal model data ^67, 68^ that demonstrate that a decrease in these anti-coagulant pathways may offset the pro-coagulant deficiency in hemophilia and promote hemostasis. Approaches with reported early phase clinical trial results include the AT siRNA therapeutic fitusiran ^53, 54^ and a monoclonal antibody directed against TFPI ^55^, concizumab; both drugs demonstrated encouraging efficacy data. The hemostatic effect of non-factor therapies (NFTs) is impervious to inhibitors. NFTs can also be administered subcutaneously at weekly to monthly frequencies, which is appealing compared to frequent intravenous administrations of standard factor products, though injection site adverse events have been reported in 15–25% of patients receiving NFTs ^49, 54^. These attributes have generated considerable excitement; however, how these new treatments will be integrated into clinical practice depends on the resolution of several ongoing debates.

### What is the risk of thrombosis from unregulated hemostasis?

In normal physiology, the endogenous pro-coagulant and anti-coagulant pathways are interwoven with multiple regulatory interactions that promote hemostasis (stopping bleeding) while minimizing thrombosis (pathological clotting). NFTs exert their hemostatic effect by circumventing these regulatory interactions in order to therapeutically “rebalance” the coagulation cascade to account for the underlying bleeding disorder ^69^. However, the new balance provided by NFTs between hemostasis and thrombosis is likely not as stable as occurs in normal physiology or with targeted factor replacement. This instability is illustrated by the observation that five of the 18 patients on emicizumab prophylaxis who experienced breakthrough bleeding and required management with aPCCs developed thrombotic complications ^49^. aPCC is a concentrate of plasma-derived zymogen and activated coagulation factors that has been used for more than four decades to treat bleeding in inhibitor patients ^70, 71^. Thrombotic complications are rare (<10 per 100,000 infusions) but are a well-recognized risk, especially when combined with other hemostatic therapies such as rFVIIa ^70, 71^. It is, therefore, not surprising that the thrombotic complications with emicizumab also occurred when emicizumab was combined with one or more additional bypassing agents. Nevertheless, the increased susceptibility of subjects receiving emicizumab and aPCC to thrombosis suggests a synergistic interplay ^72– 74^. aPCC contains FIXa ^70^, whose enzymatic activity is enhanced 20,000-fold by emicizumab ^52^ in a biochemically unregulated manner. Emicizumab, unlike FVIII, does not require activation to exert its pro-hemostatic effect, which has been suggested to result in an earlier acceleration of coagulation ^50^. As such, there is a biochemical rationale why the concomitant use of emicizumab and aPCC may be especially prothrombotic ^74^. These thrombotic complications all occurred in emicizumab prophylaxis subjects who received >100 units/kg/day of aPCC, which is within the typical dosing recommendations of aPCC (<200 units/kg/day). As FIX and FX provided by aPCC have half-lives of 18 and 40 hours, respectively, the potential for accumulation due to multiple administrations raises safety concerns. To date, the risk-mitigation strategy of limiting aPCC doses below this threshold has been successful, and emicizumab was recently approved by the FDA for HA patients with inhibitors.

The risk of thrombotic complications due to unregulated hemostasis is not unique to emicizumab. Though no thrombotic adverse events were observed in the phase I studies, elevated D-dimer levels, a marker of pathological coagulation, were noted in several study participants receiving NFTs targeting AT ^54^ and TFPI ^55^. Recently, a phase II study evaluating fitusiran was temporarily suspended after a fatal thrombotic complication ^75^. The attractive pharmacokinetic parameters of NFTs that allow for weekly to monthly dosing appear to complicate the management of breakthrough bleeding episodes, as they probably necessitate combination therapies, which will likely increase the risk of thrombotic events. How to safely combine therapies will require thoughtful consideration and empiric studies. This concern raises the question of whether hemophilia patients should be evaluated for thrombophilias prior to starting NFTs. Indeed, one of the five subjects who experienced a thrombotic complication while receiving emicizumab was heterozygous for factor V Leiden ^49^, the most common inherited thrombophilia with a prevalence of approximately 5% in the Caucasian population ^76^. It also suggests the possibility that antidotes for NFTs may be helpful in the treatment of acute bleeds, which are not available currently except for recombinant AT, which should be able to reverse fitusiran.

### Are NFTs “better” for all patients?

The possibility of prophylactic hemostatic coverage while avoiding venipuncture is probably attractive to all patients, but it remains unclear if NFTs will demonstrate better long-term outcomes than factor products for all clinical scenarios. The concerns regarding how best to treat breakthrough bleeding in inhibitor patients receiving NFTs should frame the debate about the role of ITI once NFTs enter clinical practice. ITI is challenging for patients and families; for example, almost 20% of randomized subjects withdrew from a recent ITI clinical trial ^77^. Central venous catheters are almost always required for pediatric patients and are associated with thrombotic and infectious complications ^77, 78^. However, in the absence of inhibitors, nothing works as well to control and prevent bleeding as factor replacement ^4^. Moreover, it is unlikely that NFTs will be able to provide sufficient hemostasis for major surgery or trauma necessitating combination therapy with additional bypassing agents, which are neither as efficacious nor as safe as factor replacement. As such, despite the challenges associated with ITI, successful ITI will likely continue to provide superior long-term clinical outcomes compared to NFT prophylaxis with persistent inhibitors. Better risk stratification algorithms ^4^ may identify subsets of patients who are very unlikely to tolerize with current ITI regimens, but even these patients may benefit from novel ITI strategies ^79, 80^ rather than being immediately resigned to life-long NFT prophylaxis. Whether NFT prophylaxis can be combined safely with ITI remains a critical question and requires additional studies.

The preliminary demonstrations of the efficacy of NFTs to prevent bleeding have raised the question of whether these agents will eventually supplant factor replacement as prophylaxis for patients without inhibitors. Current prophylactic regimens are challenging, with almost 40% of adult patients not routinely receiving prophylaxis ^81^. It has also been speculated that subcutaneous delivery of NFTs may allow for earlier initiation of prophylaxis in infants, which could delay factor exposure and potentially prevent inhibitor development ^82^. The previous attempt to test this hypothesis, that delaying factor exposure reduces inhibitors through the use of standard bypassing therapy, was unsuccessful owing to breakthrough bleeding ^83^, which may be mitigated by increased hemostatic efficacy of new NFTs. However, well-defined relationships between the number of factor exposures and the timing of inhibitor development ^31, 84^ support this parameter as being the most important rather than the age of exposure; indeed, emerging evidence suggests that earlier exposure to allergens may be protective ^85^. Factor exposure in the setting of immunological “danger signals”, such as traumatic bleeds or surgery, also increases the risk of inhibitor development ^86^. As it is unlikely that NFTs will provide sufficient prophylaxis for patients never to require factor products, NFTs could potentially increase the risk by concentrating exposures to situations with immunological “danger signals”. It is possible, however, that the ease of administration of NFTs will attract patients who are currently declining prophylaxis and/or who are noncompliant.

## Clinical gene therapy

Decades of collective effort on the use of adeno-associated virus (AAV) as a vector for hemophilia have culminated with recent successes in long-term expression of therapeutic FVIII and FIX levels, amelioration of the disease phenotype, and reduction or even discontinuation of factor replacement ^87– 89^. The first in-human AAV liver gene therapy for HB paved the way for the current successful strategies by showing that AAV can achieve therapeutic levels of functional FIX in a dose-dependent manner, but pre-existing neutralizing antibodies (NAb) to the AAV capsid can prevent liver transduction ^90^. These NAb are present in about 40% of the general population for some AAV serotypes ^91^. In addition, an AAV capsid-mediated cellular immune response can limit the duration of the transgene expression and is clinically recognized by an increase in liver enzymes (ALT/AST) and/or decrease in transgene expression levels ^90, 92– 94^. Subsequent trials, therefore, excluded subjects with NAb to the vector capsid and closely monitored for the capsid-directed immune response, which was managed by immunosuppression.

AAV is a single-stranded DNA, non-pathogenic, replication defective virus from the parvovirus family ^92, 95^. AAV vectors have a package capacity of 4.7 kb, which easily can accommodate FIX cDNA (approximately 1.6 kb) ^96^ but was initially challenging for FVIII cDNA (7 kb) even after the removal of the B-domain (BDD, approximately 40% of the gene, 4.4 kb) that is not required for coagulation function ^97^. Consequently, AAV-based gene therapy strategies were initially focused on HB, despite the fact that it is the least common form of hemophilia.

There are several natural AAV serotypes derived from humans and non-human primates as well as synthetic capsids, which are engineered to enhance tissue tropism ^95^. All vectors tested in recent clinical trials have high tropism for the liver, which allows delivery via peripheral vein infusion. The expression of the transgene is restricted to hepatocytes by using distinct liver-specific promoters. All studies use codon optimization to enhance transgene expression levels and, in some trials, FIX variants with enhanced biological activity.

### What are the current gene therapy clinical studies for hemophilia?

Results from recent early phase studies are summarized in Table 1. The St Jude Children’s Research Hospital and University College of London (SJCRH/UCL) HB study using AAV-8-FIX-WT at doses of 2 × 10 ^11^ to 2 × 10 ^12^ vg/kg in 10 subjects demonstrated sustained FIX levels ranging from 2–5% in a dose-dependent manner reported over a 3-year period with ongoing observations ^98, 99^. The transgene is biologically functional as observed by an approximately 90% decrease in bleeding episodes in the high-dose cohort. However, four out of six subjects presented with increased levels of ALT between weeks 7 and 10 post-vector injection, with some decrease in the FIX levels suggestive of AAV-mediated cellular immune responses. Administration of oral steroids prevented total loss of FIX expression, but prompt (<48 hours) initiation of the drug provided the best outcome. No immune responses were observed in doses ≤6 × 10 ^11^ vg/kg (n=4). In an ongoing study by Spark, an AAV-FIX variant (FIX-Padua) at a low therapeutic dose of 5 × 10 ^11^ vg/kg was delivered to 10 subjects ^100^. The hyperactive FIX-Padua is a protein with approximately eightfold higher specific activity ^101^. Thus, it was anticipated that therapeutic levels of FIX could be achieved at a dose fourfold lower than was previously associated with AAV-mediated cellular immune responses in the AAV-8 ^98, 99, 102^ and AAV-2 ^90^ trials. FIX activity reached levels of 30% without inhibitor formation to FIX-Padua, and prophylaxis with FIX concentrates was stopped in all subjects for a ≥3-month period; long-term follow-up is ongoing. Notably, the rate of immune responses to AAV was lower, as only two out of 10 subjects required immunosuppression. These results demonstrate that FIX-Padua is safe and allows therapeutic levels with a lower risk of vector capsid immune responses. Evidence of FIX-Padua as an attractive transgene was also observed in a previous study by Shire using AAV-8 for HB ^103, 104^. Long-term expression was restricted to a single subject (1 × 10 ^12^ vg/kg) with FIX activity of 20%. In this study, immunosuppression was not effective at preventing the loss of transgene expression. Again, no inhibitors to FIX-Padua were detected, as was predicted in large animal models ^105, 106^.

**Table 1.** Summary of recently reported AAV gene therapy trial results.

Sponsor	Hemophilia	Vector	Manufacturing	Dose (vg/kg)	Liver enzyme elevation †	Effective immuno- suppression?	Transgene expression (% normal)	Ref.
**SJCRH/UCL**	HB	AAV8-FIX-WT	Plasmid DNA/ mammalian cell line	2 × 10 ^11^ – 2 × 10 ^12^	4/6	Yes	2–5%	98, 99
**Spark**	HB	SPK100-FIX- Padua	Plasmid DNA/ mammalian cell line	5 × 10 ^11^	2/10	Yes	~30%	100
**Shire**	HB	AAV8-FIX- Padua	Plasmid DNA/ mammalian cell line	2 × 10 ^11^ – 3 × 10 ^12^	NR	No	0–20%	107, 108
**uniQure**	HB	AAV5-FIX-WT	Baculovirus/ insect cell line	5 × 10 ^12^ – 2 × 10 ^13^	2/5	Yes	3–12%	102
**Biomarin**	HA	AAV5-BDD- FVIII	Baculovirus/ insect cell line	6 × 10 ^12^ – 6 × 10 ^13^	7/7	Yes	19–164%	109

Abbreviations: AAV, adeno-associated virus; HA, hemophilia A; HB, hemophilia B; NR, not reported; Ref., reference; SJCRH/UCL, St Jude Children’s Research Hospital/University College of London; BDD, B-domain deleted.

† (Number of subjects in highest-dose cohort who experienced increased alanine aminotransferase)/(number of subjects in highest-dose cohort)

The uniQure study for HB is based on AAV-5-FIX-WT at doses of 5 × 10 ^12^ to 2 × 10 ^13^ vg/kg with resulting FIX levels ranging from 3–12% and nine out of 10 subjects stopped prophylaxis over 1.5 years of observation (ongoing) ^102^. However, there was not a clear dose response. An increase in ALT levels occurred in three out of 10 subjects around 10 weeks post AAV injection; all three subjects received steroids and none experienced appreciable FIX activity loss. The HA Biomarin study is based on AAV-5-BDD-FVIII. In the high-dose cohort (6 × 10 ^13^ vg/kg), sustained expression of FVIII of 19–164% of normal over a 12-month period was a rather surprising finding (ongoing) ^109^. All subjects are off prophylaxis. While an increase in ALT was documented in all subjects from this cohort, and steroids were initiated in all, there was not a clear relationship among ALT normalization, steroid use, and FVIII level stabilization. An additional safety concern raised by the AAV-5 trials is prolonged vector shedding in body fluids, including semen, where samples tested positive for vector sequences for 48 and 52 weeks (yet not cleared) post vector delivery at doses of 5 × 10 ^12^ vg/kg and 6 × 10 ^13^ vg/kg in FIX and FVIII trials, respectively. It is also important to note that age may also influence the vector shedding kinetics from the semen ^90, 110^. Results from other clinical trials have not been reported.

### What should be a feasible and safe therapeutic range?

In the early days of gene therapy, the goal was rather modest: minimal increase of factor levels above 1% could improve the severe phenotype, as noted by both natural history of non-severe disease and prophylaxis. However, in a trial with two subjects with advanced underlying joint disease, FIX levels of 1.5–3% were not sufficient to prevent bleeds, and prophylaxis was continued ^98, 99^. Thus, strategies with the potential to achieve >5% of normal are likely to be more effective, with some evidence that levels above 12% could be associated with no spontaneous bleeding ^111^. This is rather challenging for those with advanced joint disease who are likely to require surgical intervention despite these therapeutic levels.

In the general population, increased levels of FVIII or FIX are associated with increased risk of thrombosis ^112– 117^. Supraphysiological levels of FVIII in the HA study raise safety concerns ^114, 115, 118^, since men with hemophilia are not protected from cardiovascular disease and remain at risk of thrombotic complications ^118^. In addition, there is no evidence that levels above 50% of normal are associated with pathological bleeding, and hemophilia carriers have decreased mortality due to ischemic heart disease ^119^. The relative short-term follow-up of HA patients expressing elevated levels of FVIII prevent firm conclusions on the safety of this finding.

### Is codon optimization of the transgene safe?

Codon optimization uses synonymous codon changes to increase protein expression without modifying amino acid sequences. The development of numerous codon optimization programs and the commercial availability make this strategy popular. However, there are potential risks, such as the creation of alternative open reading frames and alteration in protein post-translational modifications ^120^. As such, for codon optimization, the benefits in gain of expression must be weighed against these safety concerns. To date, the biochemical characterization of a codon-optimized FVIII-BDD that is expressed sevenfold higher than the non-codon-optimized FVIII showed similar, but not identical, biological activity ^121^. The transgene used in these studies is the same as that used in the current HA trial by Biomarin ^122^.

## AAV capsid-mediated T cell responses

The most common short-term safety concern of AAV gene therapy is the generation of AAV capsid-mediated cellular responses ^90, 92– 94^. This complication is restricted to humans, as provocative preclinical studies in small and large animals failed to fully replicate the clinical findings. Encouragingly, this absence of AAV capsid-mediated cellular responses in hemophiliac dogs has resulted in >8 years of sustained expression ^123^ and created an opportunity for providing a relatively low-risk treatment to HA pet dogs ^124^. In humans, the need for rapid initiation of the immunosuppression therapy to prevent total loss of expression requires a close monitoring of the subjects, which imposes challenging clinical care as studies move beyond early phases. It is not possible to currently identify those who will develop such complications, but some clinical strategies may help to overcome it.

### What are the clinical factors triggering immune response to the vector capsid?

Data from clinical studies show that capsid-mediated immune responses occur with all serotypes in a vector dose-dependent manner. The onset and dose dependence varies among distinct serotypes. For example, in AAV-2 and AAV-8, the dose of 2 × 10 ^12^ vg/kg ^90, 99, 100^ was the threshold for immune responses, whereas for AAV-5 in one study for HB ^102, 106^ the dose was 5–10-fold higher. Interestingly, in a study using AAV-5 for HA ^105, 109, 110^, only at doses of 6 × 10 ^13^ vg/kg (30-fold higher than AAV-2 and AAV-8 cited above) were immune responses noted. The initial mechanism of elevation of liver enzymes in the trials using AAV-5 was thought to be due to AAV-capsid immune responses. However, in both studies, the dose-dependent increase in liver enzymes was not associated with detectable T cell expansion. Nevertheless, transient immunosuppression was initiated and liver enzymes normalized. The underlying mechanism of this complication is unclear at this point and may be a direct effect of the vector on the hepatocytes. Therefore, this may influence the long-term safety profile of AAV-5. One possibility for these discrepancies is that AAV-5 vectors were produced using a baculovirus system with insect cells lines, whereas, for the other serotypes, plasmid systems in mammalian cell lines were used. It is known that AAV-5 production in insect cell lines resulted in low infectivity per particle; thus, higher therapeutic vector doses were anticipated ^125– 127^. Thus, the emerging data suggest that the vector manufacturing process may also influence the safety profile of AAV. To date, a side-by-side comparison of AAV biological activity using distinct manufacturing systems is lacking. Differences in dose needed for therapeutic transgene expression between vectors may account for increased concentration of capsid that is presented to hepatocytes, leading to stimulation of memory T cells.

### Is lowering the therapeutic vector dose the safest strategy to avoid immune responses?

The study using AAV-Spark100-FIX-Padua at 5 × 10 ^11^ vg/kg resulted in sustained therapeutic levels of FIX of approximately 30% ^128^. Immune responses triggered by AAV capsid occurred in only two out of 10 subjects. In contrast, in the study using AAV-8-FIX-WT at 2 × 10 ^12^ vg/kg (fourfold higher dose), four out of six subjects developed these immune responses ^99^. Thus, the use of a transgene with enhanced biological activity allows an effective and safe strategy by minimizing vector-mediated cellular responses. This is also attractive since the AAV-5 trials showed that there is a dose-dependent elevation of the liver enzymes; thus, lowering the therapeutic dose would likely benefit all future studies.

### Is CpG content a risk factor for immune responses to the vector capsid?

Preclinical studies suggest that activation of innate immune responses through CpG-mediated Toll-like receptor 9 was a potential underlying mechanism for the stimulation of AAV capsid-mediated CD8 ^+^ T cell cytotoxicity. The use of CpG-depleted codon-optimized FIX transgene in preclinical models was aimed at high expression level ^129^. Notably, the first AAV liver trial using the same FIX transgene resulted in AAV capsid-mediated immune response in four out of six subjects in the high-dose cohort ^99^. It is, therefore, probable that the innate immune responses may differ between humans and other species and that simply removing CpGs will not prevent vector capsid immune responses in patients, though the role of the innate immune response in the anti-AAV capsid cellular immune response deserves continued study ^130^. Data on the CpG content of the other vectors have not been published.

### Is transient immunosuppression enough?

The hepatocyte toxicity triggered by AAV capsid immune responses is clinically asymptomatic and resembles, to a certain extent, autoimmune hepatitis ^131^. As such, the use of steroids as the first line of treatment is reasonable. Data suggest that steroids are most effective if initiated within 48 hours after increase of liver enzymes and/or decrease in transgene expression and should be continued for 8–12 weeks ^99, 128^. This strategy has been effective in most studies. However, in the Shire-sponsored study, therapeutic levels of FIX (peak at 60%) were achieved in the high-dose cohort (3 × 10 ^12^ vg/kg), but the duration of expression was transient and reduction in expression coincided with increased ALT ^103^. In an AAV-8-FIX clinical study at the Children’s Hospital of Philadelphia, three subjects were infused at doses of 1 to 2 × 10 ^12^ vg/kg and all developed an immune response to the vector capsid that was not controlled by immunosuppression, resulting in loss of transgene expression ^94^. Alternative immunosuppression regimens may be required ^132^.

Whether preventive therapy with steroids would be a more manageable strategy is complicated by the fact that timing of the immune response ranges from 4–10 weeks post vector delivery and depends on the vector serotype. However, it seems that, for a given serotype, the time of immune response onset is very consistent. Thus, if one subject developed immune responses, all subsequent subjects in the same dose cohort could receive prophylactic immunosuppression.

The reasons why immunosuppression can overcome immune response to the vector capsid in some, but not all, studies is unclear. It is possible that the combination of vector design (codon optimization/CpG content), the ratio of empty and full capsid particles, host-dependent factors such as HLA, and the innate ability to mount cellular immune responses could all contribute. Hopefully, as these vector systems and clinical outcomes will be further characterized in future publications, we will likely have more insights on this safety concern.

### Is there a risk of inhibitor formation to the transgene product?

As for any other novel therapy for hemophilia, there are concerns regarding the risk of inhibitor formation. To date, the presence or history of inhibitors and minimal exposure to factor concentrates are exclusion criteria in all of these studies. However, preclinical studies in small and large models showed that AAV liver-restricted expression is biased towards transgene-specific immune tolerance ^80, 133^. Moreover, in hemophiliac dogs with inhibitor to FVIII or FIX, AAV expression of the transgene was efficacious in inhibitor eradication, mimicking ITI, followed by continuing expression with improvement of the disease phenotype ^80, 105, 134^.

### Which strategy is better?

With growing numbers of clinical studies using diverse strategies, one important issue is to define a superior approach. The simplest outcome measurement, such as sustained factor levels, is a logical criterion. However, if a very high vector dose is needed to achieve high therapeutic levels, the impact on the vector manufacturing to a large patient population may be hampered by production feasibility issues and raises safety concerns. Another possibility is that of a strategy with minimal or no risk of vector-mediated immune responses yet with factor levels in the mild disease range. This could bring the benefits of prophylaxis to a large population without the time-consuming and labor-intensive monitoring of ALT and factor levels. Preventive immunosuppression could simplify this process, but, as discussed above, it is not always feasible. Furthermore, the lack of normalization on the strategy used to define the vector genome prevents direct comparison among distinct vectors ^135^.

## Closing paragraph

These new therapies will likely transform hemophilia care, providing more efficacious and convenient management options and possibly curative therapies. The largest gains will be accrued by those patients receiving only limited therapeutic benefits from current strategies, such as those with refractory inhibitors and frequent bleeding. However, it is imperative that the excitement over the considerable potential of these drugs to help undertreated patients does not obscure early safety concerns such as potential pathological PEG accumulation, thrombotic complications in NFTs, and irreversible supraphysiological factor levels after gene therapy. Furthermore, the cost of these emerging therapies is not clear; whether these new strategies will expand access to the 80% of worldwide patients who are currently not receiving regular therapy for economic reasons remains unknown. Hemophilia treaters will have to balance efficacy, convenience, price, and patient preferences and lifestyle when developing personalized treatment plans that include these novel therapies. The hemophilia community is entitled to definitive answers to these questions, which will require careful preclinical and clinical studies. As the types of available therapies become more varied, such studies must include assessments of the quality of life (QoL) of patients. The ultimate goal of therapies for hemophilia is to provide a QoL and life expectancy equivalent to those of someone without a bleeding disorder.
